# The effects of graded levels of calorie restriction: V. Impact of short term calorie and protein restriction on physical activity in the C57BL/6 mouse

**DOI:** 10.18632/oncotarget.8158

**Published:** 2016-03-17

**Authors:** Sharon E. Mitchell, Camille Delville, Penelope Konstantopedos, Davina Derous, Cara L. Green, Yingchun Wang, Jing-Dong J. Han, Daniel E.L. Promislow, Alex Douglas, Luonan Chen, David Lusseau, John R. Speakman

**Affiliations:** ^1^ Institute of Biological and Environmental Sciences, University of Aberdeen, Aberdeen, Scotland, UK; ^2^ State Key Laboratory of Molecular Developmental Biology, Institute of Genetics and Developmental Biology, Chinese Academy of Sciences, Chaoyang, Beijing, China; ^3^ Key Laboratory of Computational Biology, Chinese Academy of Sciences-Max Planck Partner Institute for Computational Biology, Shanghai Institutes for Biological Sciences, Chinese Academy of Sciences, Shanghai, China; ^4^ Department of Pathology and Department of Biology, University of Washington, Seattle, Washington, USA; ^5^ Key Laboratory of Systems Biology, Innovation Center for Cell Signaling Network, Institute of Biochemistry and Cell Biology, Shanghai Institutes for Biological Sciences, Chinese Academy of Sciences, Shanghai, China

**Keywords:** food intake, dietary restriction, protein restriction, calorie restriction, physical activity, Gerotarget

## Abstract

Calorie restriction (CR) delays the onset of age-related disease and extends lifespan in a number of species. When faced with reduced energy supply animals need to lower energy demands, which may be achieved in part by reducing physical activity (PA). We monitored changes in PA using implanted transmitters in male C57BL/6 mice in response to graded levels of CR (10 to 40%) or matched levels of graded protein restriction (PR) for 3 months. Mice were fed at lights out and *ad libitum* controls were limited to dark-phase feeding (12AL) or 24hr/day. Total daily PA declined in a non-linear manner over the first 30 days of CR or PR, remaining stable thereafter. Total daily PA was not related to the level of CR or PR. Total daily PA over the last 20 days of restriction was related to circulating leptin, insulin, tumor necrosis factor-α (TNF-α) and insulin-like growth factor (IGF)-1 levels, measured after 3 months. Mice under restriction showed a high level of activity in the 2hrs before feeding (food anticipatory activity: FAA). FAA followed a complex pattern, peaking around day 20, falling on ∼day 37 then increasing again. FAA was also positively related to the level of restriction and inversely to leptin, insulin, TNF-α and IGF-1. Non-FAA, in contrast, declined over the period of restriction, generally more so in mice under greater restriction, thereby offsetting to some extent the increase in FAA. Mice under PR displayed no changes in PA over time or in comparison to 12AL, and showed no increase in FAA.

## INTRODUCTION

Calorie restriction (CR), mainly in rodents, but also in lower animals and non-human primates, results in retardation of the aging processes and increased longevity [1-4]. However, in many studies, parallel to the restriction of calories there is a reduction in macronutrient intake, e.g. protein, leading to the suggestion that the beneficial effects of CR may be due to protein restriction (PR) [5-8]. CR leads to alterations in body composition, particularly fat loss [9-11], reductions in adipokines, lowered circulating glucose, insulin and insulin-like growth factor (IGF)-1, leading to subsequent improvements in insulin sensitivity [12-15], lowered body temperature (T_b_) [16-17] and alterations in behavior [18, 19]. Many of these physiological characteristics of CR are shared by several long lived mutant mouse models, Ames, Snell dwarf, and the growth hormone receptor/binding protein knockout mice [20-22]. In particular a lowered T_b_ and improved insulin sensitivity seem important. Experimental reduction of T_b_ by overexpressing uncoupling proteins in the brain extended lifespan [23]. In addition in rodents, lower T_b_ was responsible for tumor prevention during CR, while in humans, those with a lower T_b_ had better survival rates than those with a higher T_b_ [24, 25]. The evolutionarily conserved insulin/IGF-1 signaling pathway (IIS) is strongly implicated in CR-mediated extension of lifespan, with improved insulin sensitivity a key determinant of healthy aging [26, 27]. However while these biomarkers appear to be a pre-requisite for CR's longevity effect, the mechanisms underpinning the beneficial effects of CR remain unresolved.

To balance a restricted energy intake animals must reduce energy expenditure. There are 4 major components of daily energy expenditure: the energy cost of physical activity (PA), basal metabolic rate (BMR), thermoregulation and the thermic effect of food. BMR comprises a large proportion of energy expenditure and is dependent on both body size and body composition with the contribution of lean mass being greater than fat mass [28]. PA can be divided into spontaneous activity (also referred to as non-exercise activity thermogenesis (NEAT) and voluntary exercise [29]. Spontaneous activity constitutes obligatory survival activity, ie. food searching, while voluntary activity is not directly required for survival [30]. Available literature on the PA response to CR is contradictory. Despite the reduction in energy intake several rodent studies have shown an increase in PA levels during CR [31-34]. On the other-hand, others have reported a decrease [35, 36].

Protocol differences such as diet, genetic background, sex, duration and stringency of restriction may account for the inconsistencies found in the effect of CR on total daily PA in previous studies. For example some studies have considered only PA responses in open field tests [36], while others have measured the energy expended on PA [35], while yet others have focused on total levels of movement [36]. Responses measured by these different methods are not necessarily equivalent. However, consistent across studies is an increase in PA immediately prior to feeding each day (known as food anticipatory activity (FAA)) [37, 38]. FAA is observed in a large variety of species, including insects, fish and primates and appears to be evolutionary conserved (reviewed in [39]). The ability to be physically active and search for food is essential in the wild thus some hypothesize that FAA may be increased foraging behavior to protect against starvation and increase chances of survival [40, 41]. Increases in activity may be beneficial in the maintenance of health span by counteracting ageing. In humans, exercise evokes a number of health benefits, such as a decreased risk of cardiovascular disease, diabetes and osteoporosis *via* the maintenance of aerobic capacity, improved bone health and preservation of muscle mass and strength [42-45]. However, increased activity does not appear to be a primary factor behind CR induced longevity. While several rodent studies reported an increase in mean lifespan in response to moderate exercise, unlike CR, no extension in maximum lifespan has been reported [46, 47]. Exercise alone does not increase survival of *ad libitum* fed animals but attenuated a CR increase in animals subjected to lifelong running [48]. In addition the long-lived Ames dwarf mouse is also less active than wild type controls [49].

PA is under biological control [50] mediated *via* complex interactions between regulators of energy balance, of which leptin and insulin play a major role [51-55]. Leptin and insulin convey energy availability signals to the hypothalamus, a key site for the regulation of energy balance [56-58]. The hypothalamus not only responds to hormonal signals, *via* leptin and insulin receptors (and others) [54, 59, 60] but also directly *via* nutrient related signals, such as glucose and fatty acids (see review [61]), conveying information on available and stored energy supplies. Food availability is known to influence circadian rhythms and these alterations may contribute to the life-prolonging effect of CR [62] also see review [63]. For instance, αMUPA mice (transgenic mice overproducing the urokinase-type plasminogen activator (UPA) in many brain sites) spontaneously eat less and live longer than wildtype controls but, unlike their controls αMUPA mice can sustain circadian rhythms with age, and this may contribute to their prolonged lifespan [64].

Over a series of papers [11, 15, 17, 19] we have described the diversity of physiological, endocrine, biochemical and behavioral responses to CR, which have been compared to the responses to equivalent levels of PR [11, 15, 17]. Here we will focus on PA and FAA measured *via* non-invasive, implanted transmitters. We show that non-FAA and FAA appear to be regulated differently in relation to body composition and circulating hormones that, in turn, vary in relation to the CR manipulation.

## RESULTS

### Total daily physical activity (PA)

**Figure 1:**
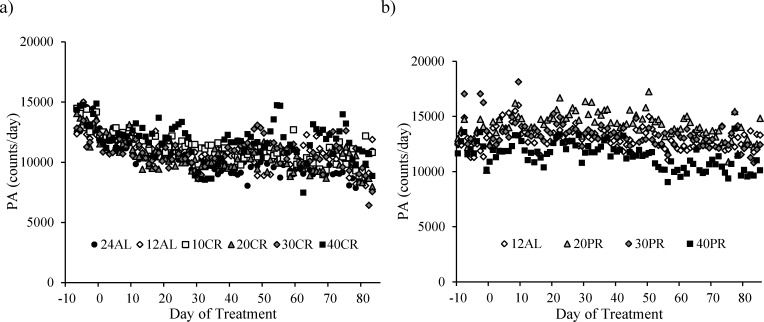
The level of total daily physical activity (PA) in mice under graded levels of a) calorie restriction (CR) or b) protein restriction (PR) The baseline period is represented as negative days −10 to −1. Day 0 denotes the start of the restriction period lasting 84 days. Data was averaged across each of the *ad libitum* (AL) groups which had access to food for either 24 or 12 hrs/day (24AL and 12AL respectively). Treatment groups were restricted in a graded manner at 10, 20, 30 and 40% groups of individual baseline food intake (10CR, 20CR, 3CR and 40CR respectively). Diets were isocaloric and protein levels in the 20PR, 30PR and 40PR matched levels of that in the 20CR, 30CR, 40CR.

**Figure 2:**
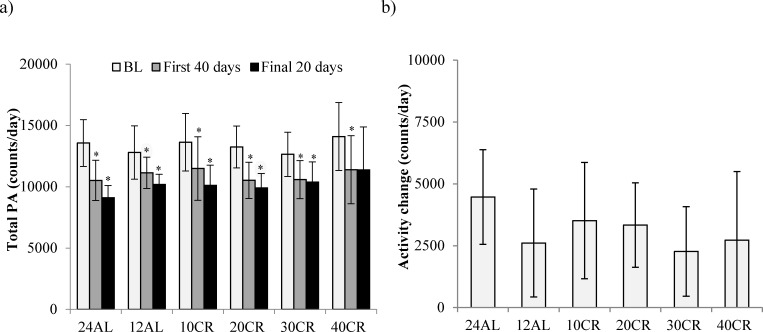
Total daily physical activity (PA) levels **a.** Total PA averaged over baseline (BL), the first 40 and the final 20 days of restriction. **b.** and the overall PA change over entire study period (b). Data shown as mean± SD. ^×^ significant lower PA compared to BL.

The level of total daily activity (counts) in relation to the baseline period and the time on CR treatment, averaged across the individuals in each of the CR groups (where food was reduced by 10, 20, 30 and 40% referred to as 10CR, 20CR, 30CR and 40CR respectively) is illustrated in Figure 1a. During the baseline period there was no significant difference in the total daily PA between the six groups (One-way ANOVA: F_(5,39)_ = 0.44, *p* = 0.816), averaging 13356 counts per day (SD = 2107, *n* = 45 individuals). Over the first 40 days of restriction daily activity was significantly decreased in all groups (paired comparison within each group: paired *t*-test: *p* < 0.05, Figure 2a). The largest reduction was observed in the animals fed *ad libitum* for 24hr (24AL) (average reduction 3046 counts, ranging +159 to −6243) and the least in the 12AL (*ad libitum* for 12hr) (1655 counts, ranging +624 to −5288). The decrease in PA over the first 40 days of restriction was curvilinear. These separate patterns averaged across the individuals for each group are illustrated in Supplementary Figure 1. The best fit (least squares) polynomial regressions to these curves were second order and the parameters of these fits with the predicted inflection points of the curves are presented in Table 1. As the severity of restriction increased, the variance explained by the fitted curves generally declined. There was no significant relationship between the time taken for the curves to reach inflection and the extent of restriction (One-way ANOVA: F_(5,36)_ = 0.21, *p* = 0.957), taking on average 31.9 (SD = 3.6) days for the curves to reach their nadirs.

**Table 1:** Parameters of fitted quadratic equations for total daily physical activity (PA)

ID	a	b	c	r^2^	int
11	2.8958	−209.10	11442	0.5245	36.104
12	5.7121	−353.37	15161	0.4791	30.932
13	7.2036	−500.16	16958	0.6575	34.716
15	1.6473	−161.44	15424	0.4023	49.001
22	4.4976	−298.95	13422	0.6574	33.234
40	2.9355	−263.00	16173	0.5366	44.796
42	−4.1486	138.73	12063	0.2274	16.720
**24AL**				**Mean**	**35.072**
16	−2.5553	102.75	11258	0.1703	20.105
17	2.9416	161.77	11570	0.2704	27.497
18	2.9993	−119.32	13808	0.1882	19.891
20	−0.9348	18.08	11538	0.1071	9.673
31	4.204	−223.80	13062	0.3036	26.618
32	3.2787	−212.01	12317	0.4117	32.331
38	1.6599	−256.02	16601	0.6452	77.119
45	0.9673	−160.87	16060	0.5826	83.154
**12AL**				**Mean**	**37.049**
8	7.1375	−413.13	12562	0.4325	28.941
9	2.7444	−196.80	10820	0.3623	35.855
21	1.3025	−138.21	12973	0.3784	53.056
33	−2.1626	64.094	13829	0.0840	14.819
46	2.9308	−189.32	15368	0.1294	32.298
50	−0.6397	11.30	13895	0.0136	8.830
54	−4.2297	120.53	13318	0.3418	14.248
56	1.6864	−208.65	18143	0.4781	61.863
**10CR**				**Mean**	**31.239**
4	3.4194	−229.39	12029	0.2937	33.542
10	2.6206	−225.54	12552	0.6111	43.032
27	0.2176	−90.71	13089	0.2460	no fit
37	3.904	−289.08	15858	0.3910	37.024
39	1.5245	−123.75	12006	0.1695	40.587
47	3.7523	−303.17	15099	0.5212	40.398
57	−0.7709	−4.29	13521	0.0918	no fit
64	7.107	−426.53	17413	0.3504	30.008
**20CR**				**Mean**	**37.432**
6	5.6075	−390.47	17006	0.4575	34.817
24	6.2294	−410.43	13377	0.6775	32.943
36	−2.449	146.88	8671	0.1947	29.988
49	1.9348	−97.92	11605	0.0407	25.305
52	−1.1278	28.75	11732	0.0459	12.748
53	4.2078	−277.57	15662	0.3773	32.983
**30CR**				**Mean**	**28.131**
7	6.8048	−552.70	19833	0.6311	40.611
28	−3.9962	122.87	12124	0.2658	15.373
30	−2.5566	−2.99	13343	0.5546	no fit
34	0.5291	−20.17	10243	0.0038	19.063
44	−1.0885	109.94	14965	0.0327	50.501
48	4.778	−349.36	12417	0.6461	36.559
58	2.1293	−148.66	5106	0.0671	34.908
62	−3.1711	64.47	12792	0.3091	10.165
**40CR**				**Mean**	**29.597**

y = a.x^2^ + b.x + c where y is PA, x is the day of restriction and a, b and c are arbitrary constants. Curves were fitted over the first 40 days of restriction. 10CR, 20CR, 30CR and 40CR signify 10, 20, 30 and 40% calorie restriction respectively. The r^2^ of the fitted quadratic equation is shown along with the calculated interpolation point in days (int). Mean interpolation points ± SD are calculated for each group.

From day 40 the patterns of change in total daily activity levels showed no significant change to the end of study (paired t-tests: *p* > 0.05, Supplementary Figure 2). In combination these changes in the total daily activity meant that activity over the last 20 days on restriction was significantly lower than the baseline levels (paired t-tests: *p* < 0.05, Figure 2a), in all groups except the 40CR group (paired t-tests: *p* = 0.128). This was principally because of a large heterogeneity in response in the 40CR group, with 5 individuals reducing activity enormously (maximum decrease 55%) while 3 increased activity (maximum increase 25%). There was no significant relationship between the extent of restriction and the change in activity over the restriction period (One-way ANOVA: F_(5,39)_ = 0.75, *p* = 0.34, Figure 2b) and no relationship between the activity averaged over the last 20 days and the level of restriction (One-way ANOVA: F_(5,39)_ = 1.14, *p* = 0.34, average 10212, SD = 1920, Figure 2a).

**Figure 3:**
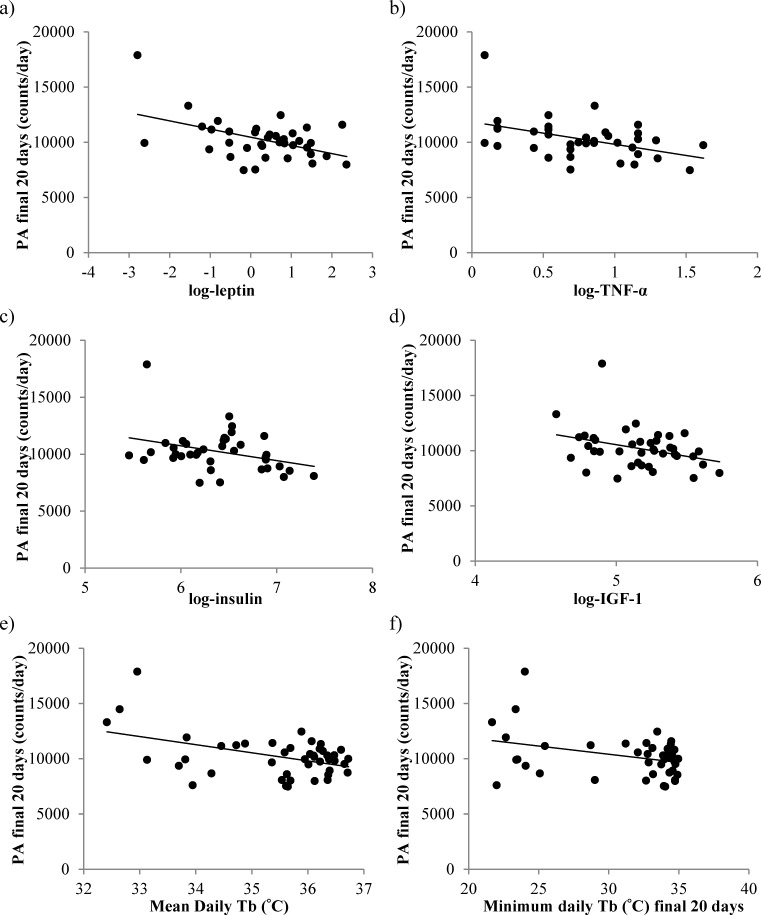
The relationship between the levels of total daily physical activity (PA), measured over the final 20 days of restriction and the levels of circulating hormones and body temperature (T_b_) **a.** leptin, **b.** tumor necrosis factor (TNF)-α, **c.** insulin, **d.** insulin growth like factor-1 (IGF-1). **e.** mean daily T_b_ and **f.** minimum T_b_. Hormone data is reported in [15] and temperature data taken from [17].

Total daily PA was not significantly related to the amount of body fat (Least squares linear regression (LSR): r^2^ = 0.072, F_(1,44)_ = 3.6, *p* = 0.065) or the levels of structural tissue (pooled mass of carcass, skin and tail; LSR: r^2^ = 0.055, F_(1,44)_ = 2.5, *p* = 0.121) (tissue data from [11]). However, there were significant associations between the final levels of activity and the final levels of circulating hormones (hormone data from [15]); leptin (LSR: negative, r^2^ = 0.232, F_(1,36)_ = 10.89, *p* = 0.002, Figure 3a), tumor necrosis factor (TNF)-α (LSR: negative, r^2^ = 0.184, F_(1,39)_ = 7.92, *p* = 0.008, Figure 3b), insulin (LSR: negative, r^2^ = 0.104, F_(1,36)_ = 4.19, *p* = 0.048, Figure 3c) and IGF-1 (LSR: negative r^2^ = 0.097, F_(1,39)_ = 4.10, *p* = 0.05, Figure 3d), but not interleukin (IL)-6 (LSR: r^2^ = 0.025, F_(1,36)_ = 0.91, *p* = 0.347) or resistin (LSR: r^2^ = 0.072, F_(1,36)_ = 0.26, *p* = 0.612). When all the available hormone levels were entered as predictors in a multiple regression analysis, both leptin and IL-6 were found to be significant predictors of the level of activity (combined r^2^ = 0.330, leptin, *p* < 0.001 negative, IL-6 *p* = 0.03 positive). Moreover, across all the individuals the average levels of activity were significantly negatively related to the average daily T_b_over the last 20 days (LSR: r^2^ = 0.180, F_(1,43)_ = 10.69, *p* = 0.002, Figure 3e) and the minimum T_b_ over the same final 20 day period (LSR: r^2^ = 0.112, F_(1,43)_ = 5.42, *p* = 0.025, Figure 3f) (temperature data from [17]).

**Figure 4:**
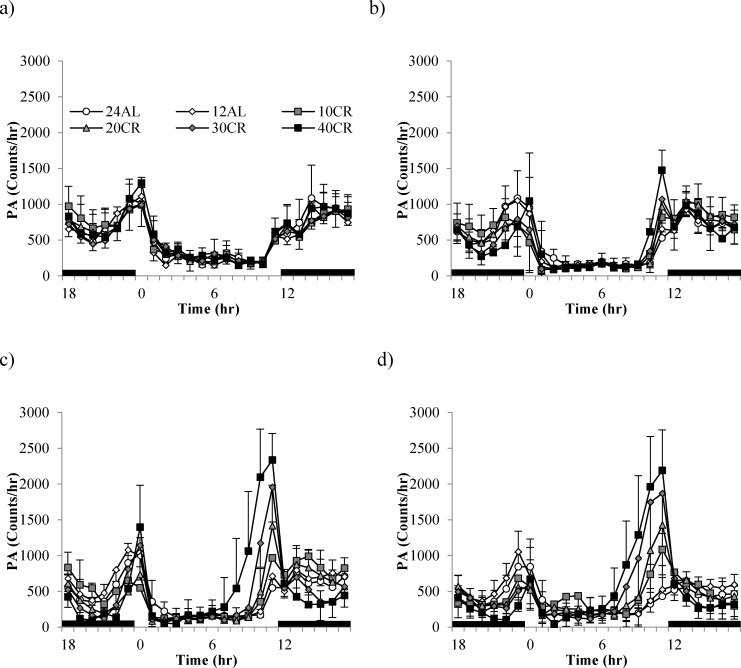
Temporal patterns of physical activity (PA) over a 24hr period at 4 timepoints over 12 weeks of calorie restriction (CR) **a.** baseline, **b.** 1 week of CR **c.** 4 weeks of CR and **d.** at the end of study following 11 weeks of CR. Black bars along the x-axis indicate the period of darkness (12-24hrs). Lights are on from 0-12 hrs. CR mice were fed at lights out. 24AL and 12AL represent animals fed *ad libitum* for 24 and 12hrs respectively. The 4 treatment groups restricted by 10, 20, 30, 40% are referred to as 10CR, 20CR, 30CR and 40CR respectively. Data is presented as mean ± SD.

Despite similar levels of total daily PA between the diet groups there was a high variation in time-based patterning over the course of the study (GLM-RM: time F_(3,39)_ = 22.94, *p* < 0.0005; Figure 4). Diet or an interaction with time were not significant (F_(3,39)_ = 0.447, *p* = 0.813 and F_(3,39)_ =1.03, *p* = 0.427, respectively). During the baseline period, when all animals were allowed AL access to food only in the hours of darkness (lights on 0630 (0hr), lights off 1830 (12hr), animals followed very similar activity patterns, ie. more active during the dark phase than during the light (Figure 4a). An increase in activity corresponded to the time when mice were weighed and fed prior to lights out (12hr). Activity remained high throughout the period of darkness with a slight lull between 18 and 23hr. A second rise in activity ∼ 22hr corresponded to lights on and food removal. Once the lights came back on (0hr) the animals reduced their activity and this low level of activity remained through the rest of the light period. As early as week 1 (Figure 4b) changes in circadian patterns were evident. An increased peak in activity prior to feeding and lights out was recorded in the CR groups, specifically the 40CR (2 fold increase from 735 to 1475). Following food consumption, activity levels dropped to that of baseline levels over the dark phase, rising again ∼ 3 hours prior to lights on. By week 4, corresponding to the time taken to reach inflection, the pattern of activity in the CR animals was very different with arousal evident in the light phase ∼ 7-8hr (Figure 4c). Activity reached peaks of ∼2500 counts/hr in the 40CR mice (>3 fold higher than baseline). A clear gradation in these PA patterns was exhibited in the 30CR, 20CR and 10CR mice reaching peaks of 1957, 1416 and 966 counts/hr respectively. In addition, animals under CR dropped their levels of activity in the late dark phase to that of daytime activity, rising again prior to lights on 0hr. Over the 12 weeks of study the daily activity patterns remained similar to that of baseline in the 24AL and 12AL animals but the pattern in the CR animals was very different (Figure 4d). The level of activity declined to the same level as the AL animals once the food was delivered. When the lights are switched on all animals reduced their activity, but around 7-8hrs, when the lights were still on, the activity started to increase. Between 10 and 12hrs, at which time the food was delivered, and the lights were switched off, the mice in the CR groups showed intense activity, higher than at any other time. The intense activity, 2-3 hrs prior to the food being delivered, has been observed previously in mice under restricted feeding protocols [65, 66] and known as food anticipatory activity (FAA). We calculated FAA as the number of counts that occurred in the 2hr prior to lights out. We then subtracted this from the total daily activity and called this remainder non-FAA. We analyzed these two types of sub-activity separately.

### Food anticipatory activity (FAA)

**Figure 5:**
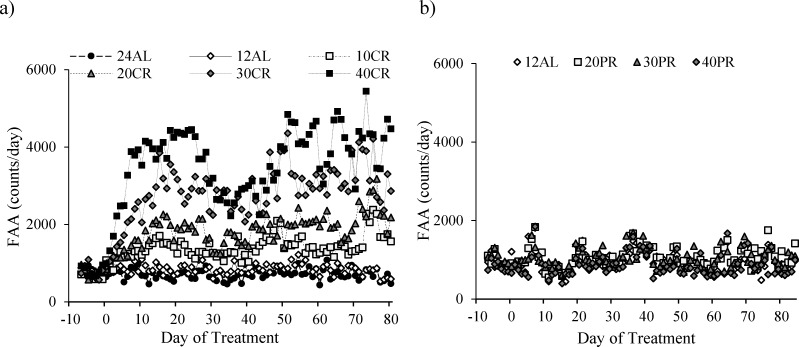
Food anticipatory activity (FAA) measured over entire study in mice under graded levels of a) calorie restriction (CR) or b) protein restriction (PR) Days −10 to −1 indicate the baseline period when all mice were fed *ad libitum* for 12 hours of dark phase (12AL). D0 represents the start of the restriction period where treatment groups were restricted by 10, 20, 30 and 40% of individual baseline food intake (10CR, 20CR, 3CR and 40CR). An additional AL group received food 24 hours (24AL). Protein levels in the 20PR, 30PR and 40PR matched that of 20CR, 30CR, 40CR. Data was averaged over each group.

**Figure 6:**
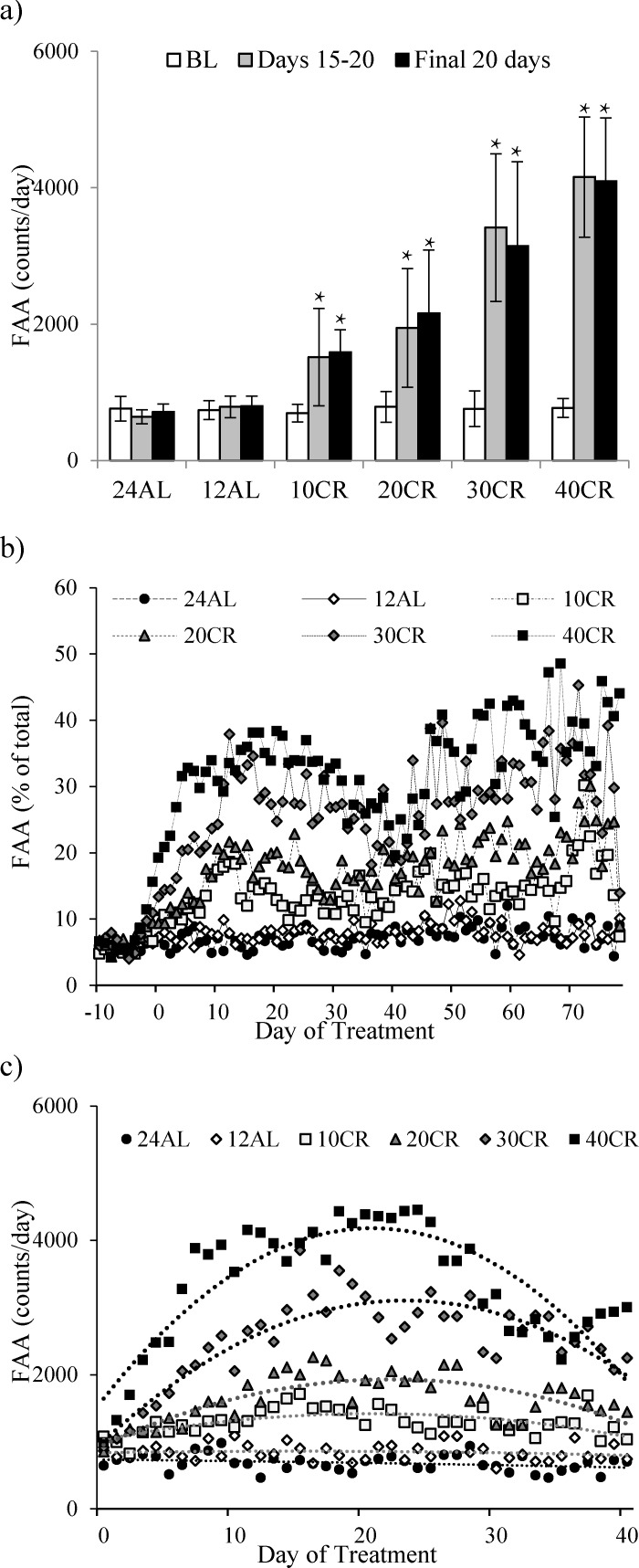
Food anticipatory activity (FAA) measured in C57BL/6 mice under graded levels of calorie restriction (CR) **a.** FAA at baseline (BL), Days 15-20 and the final 20 days of the study, **b.** The % contribution on FAA (averaged over the 2 hrs prior to feeding) to that of total daily physical activity, **c.** FAA over first 40 days of CR. 10CR, 20CR, 30CR and 40CR represent groups restricted at graded levels of 10, 20, 30 and 40%. 12AL and 24AL represent 12 and 24 hr *ad libitum* fed groups. Data presented as mean ± SD with ^×^ denoting significant increases in FAA compared to BL.

During the baseline period mice averaged 753 (SD = 173) counts during the 2 hours prior to lights out which was on average 5.7% (SD = 1.2) of their total daily activity. There was no significant difference between the treatment groups at this stage (One way ANOVA: F_(5,39)_ = 0.24, *p* = 0.942). FAA started to increase immediately after the treatment started in the CR groups, but not in those under 24AL and 12AL (Figure 5a). By days 15-20 the increase had stabilized and there was a highly significant relationship between the extent of restriction and the level of FAA (One way ANOVA: F_(5,39)_ = 28.93, *p* < 0.0001; Figure 6a).

For mice under AL feeding very little change in FAA was recorded after baseline (Figure 6a). In the CR groups FAA at day 15-20 was increased by a factor of 2.2 in the 10CR group, 2.5 in the 20CR animals, 4.9 in the 30CR group and 5.5 fold in the 40CR mice (paired t-tests: p < 0.05, Figure 6a). Consequently the percentage of the total daily activity that occurred during the 2hr period before lights out increased from 5.7% during baseline in all groups, to 14.7% at 10CR, 19.1% at 20CR, 31.4% at 30CR and 35.9% at 40CR respectively, between days 15 to 20 (Figure 6b). FAA of the two AL groups was 6.0 and 7.0% over the same time period.

After day 20 the level of FAA declined (Figure 5a) and hence the % contribution of this period to total daily activity also declined (Figure 6b). The extent of decline was related to the level of restriction, being greater in the more restricted groups, and was absent in both the groups of AL treated animals. This reduction reached a nadir around day 37, after which the levels of FAA increased again. Over the first 37 days the patterns of FAA were best modelled by quadratic second order polynomials (Figure 6c). The fits and calculated inflection points for these fitted curves are in Table 2. The calculated inflection points in the FAA occurred between 21 and 27 days after restriction started, irrespective of the level of restriction. Between days 50 and the end of the experiment the level of FAA was stable and returned approximately to the level that was observed between days 15 and 20 (Figure 5a and 6a). Because there was a reduction in total activity between days 15-20 and the end of the experiment (Figure 1a) the % contributions of FAA to the total were slightly increased over the last 20 days when compared to days 15-20 and were on average 7.6% at 24AL, 7.3% at 12AL, 16% at 10CR, 20.9% at 20CR, 32.4% at 30CR and 38.6% at 40CR (Figure 6b). These levels were highly significantly different between treatment groups (One way ANOVA: F_(5,39)_ = 25.45, *p* < 0.0005).

**Table 2:** Parameters of fitted quadratic equations for food anticipatory activity (FAA)

ID	a	b	c	r^2^	int
11	0.071	−6.29	759.52	0.012	44.27
12	0.290	−24.20	1051.70	0.177	41.68
13	0.053	−6.12	655.89	0.053	58.30
15	−0.438	16.00	820.40	0.080	18.25
22	−0.265	8.06	650.52	0.064	15.21
40	−0.070	1.50	803.85	0.007	10.70
42	0.357	−10.70	645.68	0.124	15.00
**24AL**				**Mean**	**29.06**
16	−0.673	32.72	490.71	0.260	24.30
17	−0.072	1.40	842.99	0.010	9.75
18	−0.521	31.07	569.28	0.098	29.84
20	−0.566	34.10	527.63	0.119	30.12
31	−0.108	5.15	751.28	0.003	23.78
32	−0.060	1.82	681.93	0.007	15.14
38	0.224	−11.11	788.83	0.028	24.83
45	0.229	−13.14	1171.50	0.012	28.75
**12AL**				**Mean**	**23.31**
8	−1.215	69.61	329.86	0.317	28.64
9	−2.163	118.71	260.18	0.444	27.44
21	−0.362	21.25	624.58	0.053	29.34
33	−0.464	32.78	703.73	0.230	35.33
46	−0.443	34.17	504.84	0.192	38.53
50	−0.307	21.38	661.80	0.072	34.82
54	−1.587	92.18	617.72	0.285	29.05
56	−2.016	104.44	528.94	0.332	25.91
**10CR**				**Mean**	**31.13**
4	−0.224	27.98	626.68	0.348	62.53
10	−0.766	54.56	356.92	0.431	35.60
27	−2.385	139.27	512.90	0.377	29.20
37	−0.694	35.87	1173.70	0.092	25.86
39	−0.979	52.39	458.70	0.242	26.75
47	−0.841	52.12	536.00	0.228	31.00
57	−3.422	203.37	−155.53	0.600	29.71
64	−3.720	211.59	−134.16	0.643	28.44
**20CR**				**Mean**	**33.64**
6	−2.824	184.12	695.50	0.386	32.60
24	−1.976	121.09	156.59	0.428	30.64
36	−4.626	271.50	−592.30	0.593	29.35
49	−3.392	209.95	−148.00	0.525	30.95
52	−3.656	250.68	−503.29	0.603	34.28
53	−1.464	94.94	381.12	0.548	32.43
**30CR**				**Mean**	**31.71**
7	−4.620	261.34	134.65	0.504	28.28
28	−5.646	318.78	18.51	0.473	28.23
30	−5.471	327.29	−473.36	0.606	29.91
34	−5.577	316.51	−629.11	0.506	28.38
44	−6.017	353.38	−585.35	0.519	29.37
48	−3.227	182.83	52.63	0.696	28.33
58	−5.993	344.46	−530.43	0.534	28.74
62	−6.562	353.74	−409.20	0.727	26.95
**40CR**				**Mean**	**28.52**

y = a.x^2^ + b.x + c where y is FAA, x is the day of restriction and a, b and c are arbitrary constants. Curves were fitted over the first 40 days of restriction. 10CR, 20CR, 30CR and 40CR signify 10, 20, 30 and 40% calorie restriction respectively. The r^2^ of the fitted quadratic equation is shown along with the calculated interpolation point in days (int). Mean interpolation points are calculated for each group.

**Figure 7:**
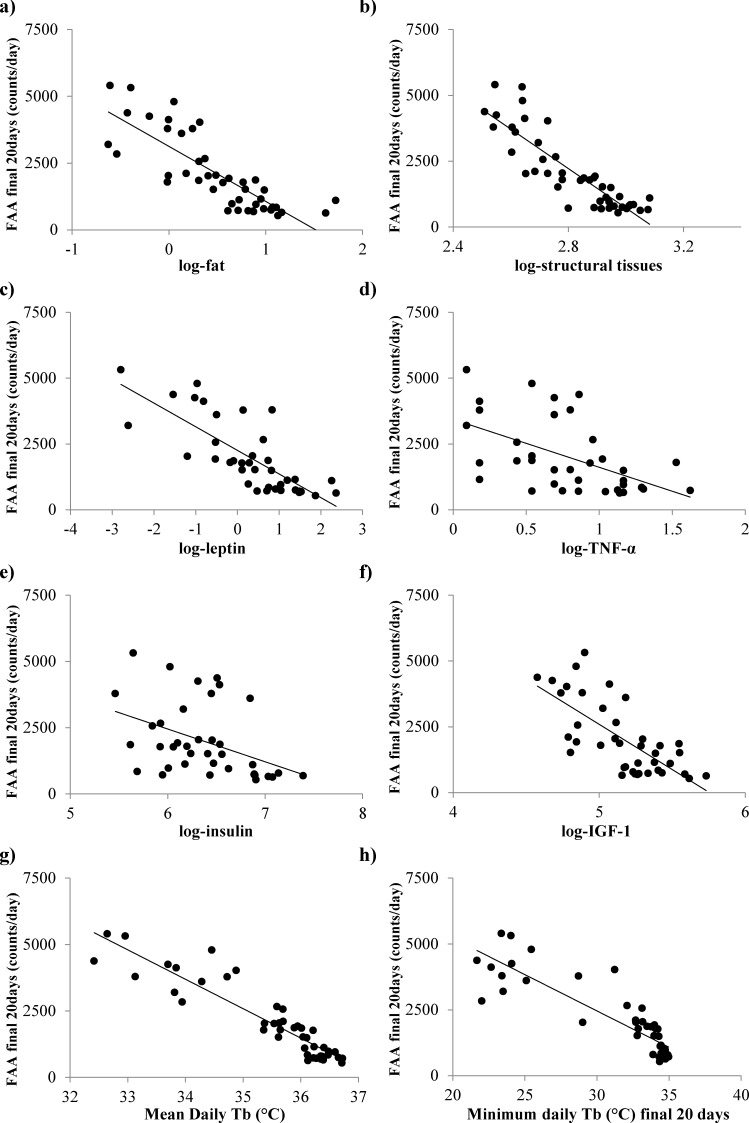
Significant associations between the levels of food anticipatory activity (FAA) averaged over the final 20 days of restriction and body composition, levels of circulating hormones and body temperature (T_**b**_) **a.** body fat, **b.** structural tissues, **c.** leptin, **d.** tumor necrosis factor (TNF)-α, **e.** insulin, **f.** insulin growth factor (IGF)-1, **g.** mean daily T_b_ and **h.** minimum T_b_. Body composition data from [11], hormone data from [15] and T_b_ data from [17].

At the individual level final FAA levels were significantly negatively related to the total body fatness (LSR: r^2^ = 0.672, F_(1,43)_ = 88.68, *p* < 0.0005, Figure 7a) and the amount of structural tissue (carcass, skin and tail) at the end of the experiment - day 90 of CR treatment (LSR: r^2^ = 0.760, F_(1,43)_ = 136.18, *p* < 0.0005, Figure 7b) (tissue weights from [11]). In a multiple regression analysis including all the individual organ weights as predictors the only significant predictors of FAA were the carcass weight (*p* < 0.0005) and the mass of the brown adipose tissue (positive association: T = 2.48, *p* = 0.02). There were also significant associations (negative) between the levels of FAA over the final 20 days and the final levels of circulating hormones (hormone data from [15]: leptin (LSR: r^2^ = 0.602, F_(1,36)_ = 54.53, *p* < 0.0005, Figure 7c), TNF-α (LSR: r^2^ = 0.286, F_(1,39)_ = 14.04, *p* = 0.001, Figure 7d), insulin (LSR: r^2^ = 0.179, F_(1,36)_ = 7.87, *p* = 0.008, Figure 7e) and IGF-1 (LSR: r^2^ = 0.513, F_(1,39)_ = 40.1, p < 0.0005, Figure 7f), but not with IL-6 (LSR: r^2^ = 0.001, F_(1,36)_ = 0.04, *p* = 0.848) or resistin (LSR: r^2^ = 0.007, F_(1,36)_ = 3.00, *p* = 0.092). When all the measured hormone levels were entered as predictors in a multiple regression analysis both leptin and IGF-1 entered as significant predictors (both negative) of the level of FAA (combined r^2^ = 0.690, leptin *p* < 0.001, IGF-1 *p* = 0.003). Average and minimum T_b_ over the final 20 days was strongly negatively correlated with FAA (LSR: r^2^ = 0.853, F_(1,43)_ = 248.94, *p* < 0.0005, Figure 7g and r^2^ = 0.769, F_(1,36)_ = 143.57, *p* < 0.0005, Figure 7h), respectively.

### Non-FAA

**Figure 8:**
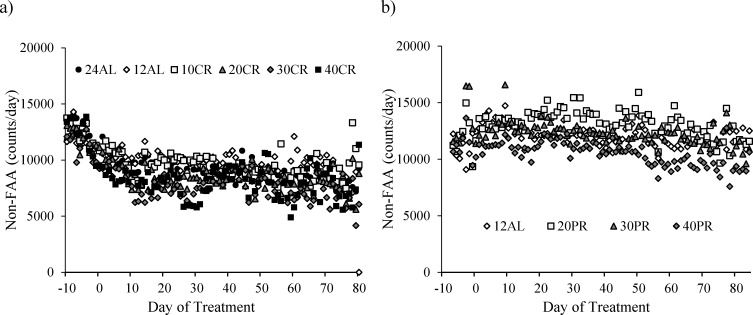
The level of non-food anticipatory activity (non-FAA) in mice under graded levels of a) calorie restriction (CR) or b) protein restriction (PR) Negative days −10 to −1 indicate the baseline period while Day 0 denotes the start of the restriction period. Data was averaged across each of the *ad libitum* (AL) groups which had access to food for either 24 or 12 hrs/day (24AL and 12AL respectively). Treatment groups were restricted in a graded manner at 10, 20, 30 and 40% groups of individual baseline food intake (10CR, 20CR, 3CR and 40CR). Protein levels of the 20PR, 30PR and 40PR matched that in the 20CR, 30CR, 40CR.

**Figure 9:**
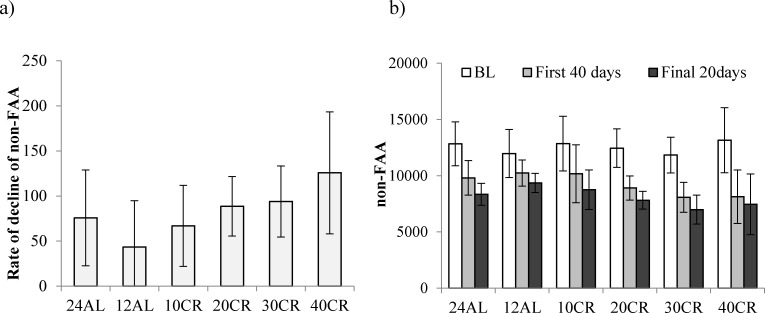
Non-food anticipatory activity (non-FAA) in mice under graded levels of calorie restriction (CR) **a.** the rate of decline of non-FAA and **b.** non-FAA measured at baseline (BL), the first 40 and the final 20 days of restriction. Non-FAA was calculated from subtraction of FAA from total daily physical activity. Restriction levels were set at 10%, 20%, 30% and 40% (10CR, 20CR, 3CR and 40CR respectively). 12AL and 24AL represent 12hr and 24hr *ad libitum* fed groups. Data shown as mean ± SD and ^×^ shows significant difference in comparison to BL levels of non-FAA.

Subtracting the levels of FAA from the total activity allowed us to investigate the changes in the non-FAA component of the total activity, and therefore to explore whether mice under restriction and demonstrating increased levels of FAA compensated this increase by decreasing their activity at other times of the day. During the baseline period non-FAA comprised the majority of activity in all animals, averaging 94.3% of the total. The patterns of change in non-FAA are shown in Figure 8a. Non-FAA showed a strong curvilinear decrease over the first 30-40 days of restriction, after which the levels stabilized. We fitted quadratic polynomials to the data for the 7 days of baseline and first 40 days of treatment for all individuals where this was possible (1 individual in the 12AL and 2 in the 10CR group had non-significant changes and no fit could be made) and calculated the initial rate of decline (at day 0) and the time to inflection of the curves. There was no significant impact of the level of restriction on the time to inflection of the fitted curves (One-way ANOVA: F_(1,39)_ = 2.17, *p* = 0.080, Figure 8a). The mean time for the curves to reach a nadir was 35.8 days (SD = 13.7, *n* = 41). However, mice under greater levels of restriction had a significantly faster rate of initial decline in non-FAA (One-way ANOVA: F_(1,39)_ = 2.45, *p* = 0.05, Figure 9a). Hence the initial rate of decline in the 12AL group averaged 43 counts per day while in the 40CR group it was almost 3 times higher at 126 counts (post-hoc Tukey *p* < 0.022). Despite differences in rate of decline, the average level of non-FAA over the first 40 days of the treatment was similar between groups (One-way ANOVA: F_(1,39)_ = 2.45, *p* = 0.074) and remained similar over the last 20 days of study (One-way ANOVA: F_(1,39)_ = 2.27, *p* = 0.067, Figure 9b).

**Figure 10:**
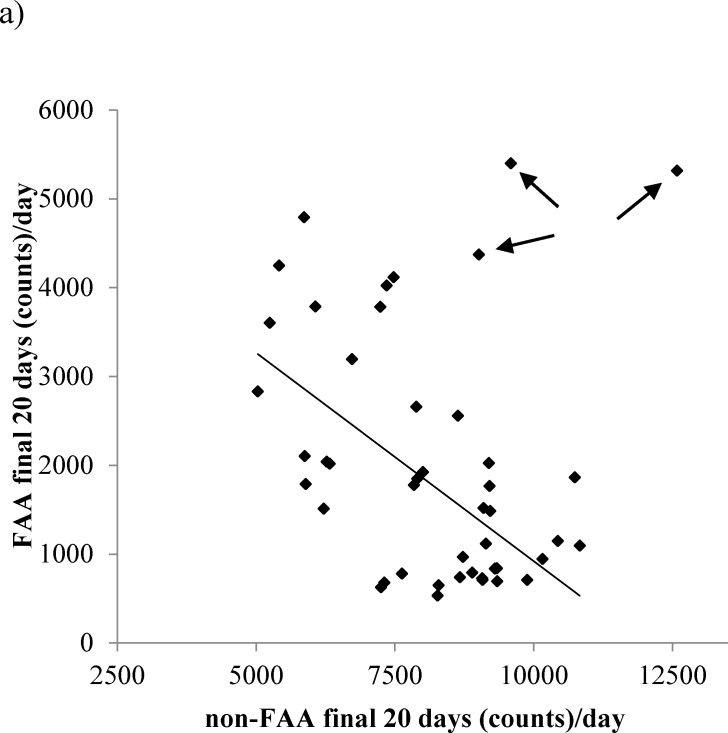
The relationship between non-food anticipatory activity (non-FAA) and FAA over the final 20days of restriction The trend line symbolizes the strong negative relationship between non-FAA to FAA when 3 individuals from 40CR group with high non-FAA and FAA (indicated by arrows) were removed.

Non-FAA over the last 20 days of the experiment was related positively to the final BM of the mice (LSR: r^2^ = 0.016, F_(1,43)_ = 8.52, *p* = 0.006) specifically, body fat (LSR: r^2^ = 0.129, F_(1,43)_ = 6.4, *p* = 0.015) and structural tissues (LSR: r^2^ = 0.205, F_(1,43)_ = 11.08, *p* = 0.002). There was no significant relationship between the level of non-FAA over the last 20 days of the experiment and the circulating levels of leptin (LSR: r^2^ = 0.018, F_(1,36)_ = 0.66, *p* = 0.421), TNF-α (LSR: r^2^ = 0.003, F_(1,35)_ = 0.01, *p* = 0.913), insulin (LSR: r^2^ = 0.02, F_(1,36)_ = 0.01, *p* = 0.928), IGF-1 (LSR: r^2^ = 0.006, F_(1,39)_ = 2.43, *p* = 0.128), IL-6 (LSR: r^2^ = 0.048, F_(1,36)_ = 1.81, p = 0.187) or resistin (LSR: r^2^ = 0.085, F_(1,36)_ = 0.31, *p* = 0.582). Across all the individuals there was no significant relationship between the level of FAA and the level of non-FAA (LSR: r^2^ = 0.055, F_(1,43)_ = 2.49, *p* = 0.122, Figure 10). The absence of a significant relationship was heavily influenced by three mice from 40CR group that had both high non-FAA and FAA (as indicated by arrows in Figure 10). If these three data were excluded then among the remaining individuals there was a strong negative relationship of non-FAA to FAA, when both were averaged over the last 20 days of the experiment (r^2^ = 0.37, F_(1,40)_ = 23.52, *p* < 0.0005).

### Protein restriction (PR)

#### Total daily physical activity (PA)

**Figure 11:**
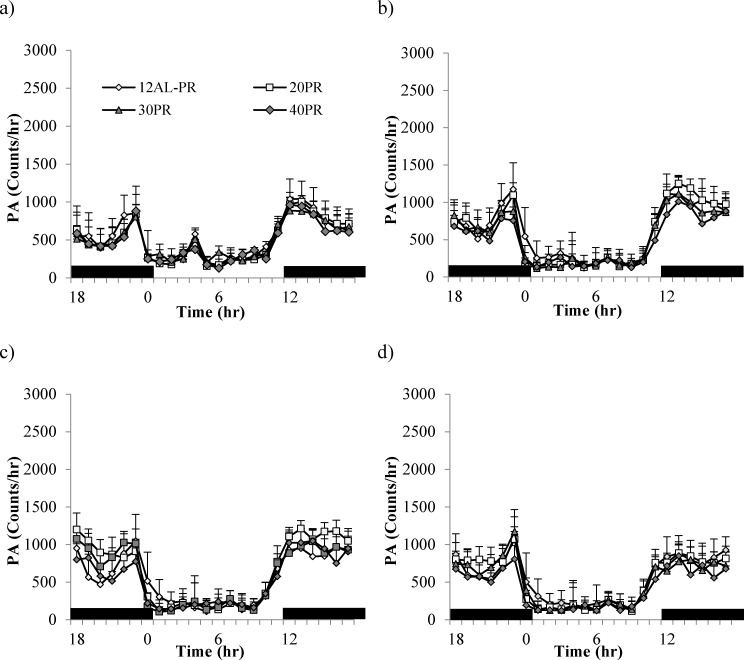
Physical activity (PA) shown over a 24hr period at 4 timepoints over 12 weeks of protein restriction (PR) **a.** baseline, **b.** 1 week **c.** 4 weeks and **d.** and 11 weeks of PR. Mice were fed at lights out (12hr) as indicated by black bars along the x-axis. 12AL represent animals fed *ad libitum* for 12hrs. 20PR, 30PR and 40PR refers to animals were diet was restricted by 20, 30, 40% protein without the reduction in calories. Data is presented as mean ± SD.

There were no differences is total daily PA between any of the groups over the baseline period, during which time all mice were fed the same diet (average 12986 counts/day, SD = 1901; One-way ANOVA: F_(3,10)_ = 0.59, *p* = 0.635, Figure 1b and Table 3). The level of total daily PA did not change over the course of the PR treatment with no evidence of an impact of PR (GLM-RM: time F_(1,14)_ = 0.670, *p* = 0.427, diet F_(1,14)_ = 0.284, *p* = 0.603, or interaction between the two factors F_(1,14)_ = 0.346, *p* = 0.566, Figure 1b). The hourly patterns of PA over the course of the study are shown in Figure 11. All groups of animals displayed very clear and comparable, nocturnal activity, low over the light phase, rising prior to lights out, at which time the mice are weighed and fed. A very similar pattern was evident over the 4 time points analyzed; the baseline period (Figure 11a and Table 3a), after 1 week of treatment (Figure 11b), after 4 weeks of treatment (Figure 11c) and at the end of the study (Figure 11d and Table 3a).

**Table 3:** Average daily physical activity (PA), food anticipatory activity (FAA) and non-FAA in animals under protein restriction (PR)

Diet	Total-PA		FAA		Non-FAA	
	BL	FL	BL	FL	BL	FL
**12AL**	12405 ± 158	12592 ±1827	875 ± 101	910 ± 137	11307 ± 263	11668 ± 1699
**20PR**	13117 ± 2238	13312 ± 2563	937 ± 247	1093 ± 254	12147 ± 2152	12140 ± 2464
**30PR**	14149 ± 2920	12678 ± 1584	1052 ± 166	1017 ± 276	13177 ± 2928	11582 ± 1653
**40PR**	12186 ± 1175	10511 ± 872	816 ± 71	860 ± 105	11302 ± 1137	9532 ± 1025

PR was graded at 20, 30 and 40% (20PR, 30PR and 40PR respectively). PR was started at 20weeks old for the duration of 3 months. Data shown as average ± SD counts /day and counts /hr for FAA. No significant differences were found between groups at any time point or within groups when baseline (BL) values were compared to final values at the end of the study (FL).

**Figure 12:**
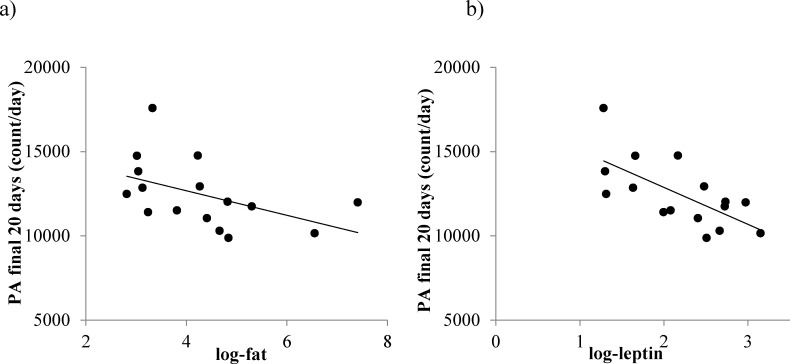
The relationship between total physical activity (PA), fat mass and circulating leptin levels in mice where protein levels were restricted by 20%, 30% or 40% (20PR, 30PR and 40PR) **a.** total daily PA *vs* fat and **b.** total daily PA and leptin. Fat mass data was taken from [11] and leptin data from [17].

Total daily PA over the last 20 days of the experiment was related to body fat (LSR: r^2^ = 0.252, F_(1,14)_ = 4.71, *p* = 0.048, Figure 12a) but not the structural tissues (LSR: r^2^ = 0.193, F_(1,14)_ = 3.35, *p* = 0.088) or vital organs (LSR: r^2^ = 0.224, F_(1,14)_ = 4.05, *p* = 0.064). A significant relationship was found between total daily PA over the last 20 days and leptin (LSR: r^2^ = 0.449, F_(1,14)_ = 11.22, *p* = 0.005, Figure 12b) but not insulin (LSR: r^2^ = 0.257, F_(1,14)_ = 4.14, *p* = 0.064) or IGF-1 (LSR: r^2^ = 0.116, F_(1,14)_ = 1.83, *p* = 0.197). In addition, total daily PA was not associated with average body temperature recorded at the end of study (LSR: r^2^ = 0.008, F_(1,14)_ = 0.12, *p* = 0.734). A summary of all relationships is shown in Table 4.

**Table 4:** Morphological and hormonal relationships between average daily physical activity (PA), food anticipatory activity (FAA) and non-FAA in animals under protein restriction (PR)

Diet	Total-PA		FAA		Non-FAA	
	T	p	T	p	T	p
**FM**	−2.17	0.048	−0.01	0.990	−0.01	0.992
**St.Tiss**	−1.83	0.088	0.31	0.761	−0.06	0.957
**Leptin**	−3.35	0.005	−1.14	0.272	−1.09	0.298
**Insulin**	−2.04	0.064	−2.64	0.021	−1.11	0.293
**IGF-1**	−1.35	0.197	0.12	0.909	0.62	0.544
**Av Tb**	−0.35	0.734	−0.94	0.366	−0.53	0.608

PR was started at 20weeks old for the duration of 3 months with PR graded at 20, 30 and 40% (20PR, 30PR and 40PR respectively). FM = fat mass, St.Tiss = structural tissues, IGF-1 = insulin-like growth factor 1, Av Tb = average body temperature.

### Food anticipatory activity (FAA)

No differences in FAA counts, averaged over the baseline period, were observed between all groups or in comparison to FAA averaged over the final 20 days of study (paired *t*-test: t_15_ = 2.15, *p* < 0.051, Figure 5b). The change in FAA ranged from +2 to −298, comprising 7.1 and 8% of total activity at baseline and the final 20 days of study, respectively. The level of FAA over the final 20 days of study was not related to body composition or temperature data. A significant relationship was noted with FAA and insulin levels measured at end of PR (LSR:*r*^2^ = 0.329, F_(1,13)_ = 7.21, *p* = 0.021). Data for FAA counts/hr at baseline and end of study are shown in Table 3 with relationship data shown in Table 4).

### Non-food anticipatory activity (Non-FAA)

No change in non-FAA was apparent between the 12AL and PR groups over the duration of study (paired t-test: t_15_ = 2.07, *p* = 0.058, Figure 8b). Non-FAA was not related to the FAA and no relationships between non-FAA and the sizes of the different tissues or circulating hormone levels were apparent (see Table 3 and Table 4).

### Comparison of responses to calorie and protein restriction (CR *vs* PR)

**Figure 13:**
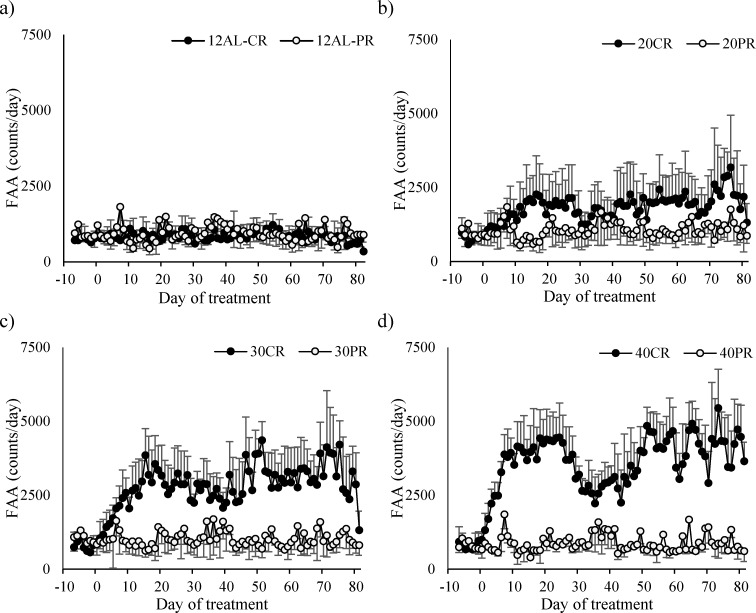
Comparisons of the food anticipatory activity (FAA) responses to caloric restriction (CR) and protein restriction (PR) FAA in **a.** control *ad libitum* fed mice *(*12AL) from both studies fed the same diet (20% protein) available only during the hours of darkness. **b.**, **c.** and **d.** show FAA response of the mice under 20, 30 and 40% CR matched to mice under 20, 30 and 40% PR respectively. In each of these instances the level of protein was the same. All plots are presented on a common scale. The x-axis is the day of measurement. Day 0 is the start of restriction. Prior to that (negative days) all mice were at baseline and fed only during the 12 hours of darkness. (*n* = 8 individuals per group except in 30CR where *n* = 6). Data is presented as mean ± SD.

With both the CR and PR experiments containing identically treated 12AL groups fed diets consisting 20% protein, comparisons were made to evaluate whether a reduction in calories or protein impacted on the activity of restricted animals. Comparing the two 12AL groups from the PR and CR studies, no differences were noted in total daily PA, FAA (Figure 13a) or non-FAA over the baseline period (2 sample t-tests: t_9_ = 0.5, *p* = 0.632, t_9_ = −1.777, *p* = 0.136, and t_9_ = 8.62, *p* = 0.414 respectively). Direct comparisons between the three PR groups and the protein level matched CR group also found no difference in PA, FAA or non-FAA over the baseline period, ie 20CR *vs* 20PR, 30CR *vs* 30PR and 40CR *vs* 40PR (2 sample t-tests: p > 0.05).

Looking at the activity over the final 20 days of study, the 12AL group from both CR and PR studies behaved in a very similar manner for total daily PA and FAA (2 sample t-tests: t_10_ = −2.507, *p* = 0.066, t_10_ = −1.266, *p* = 0.234) but not non-FAA, which was lower in the 12AL group from the CR study (t_10_ = −3.218, *p* = 0.009). Total daily PA at the end of the study was significantly higher in the animals fed 20PR (13312, SD = 2563) than those fed 20CR (9906, SD = 1171) (2 sample t-test: t_11_ = −3.308, p = 0.007). The total daily PA of the 30CR *vs* 30PR and 40CR *vs* 40PR were not significantly different (2 sample t-tests: t_8_ = −1.943, *p* = 0.088 and t_9_ = 0.410, *p* = 0.691 respectively).

However analysis of FAA found the protein matched CR and PR treated animals responded very differently. Pronounced differences were found in FAA over the final 20 days between the groups matched for the levels of protein intake ie average FAA at end of study was twice as high in the 20CR compared to 20PR (2159, SD = 928 *vs* 1093, SD = 254 counts; 2 sample t-test: t_11_ = 2.476, *p* = 0.031, Figure 13b). This was amplified as the restriction levels increased and FAA was 3 times greater in 30CR (3144, SD = 1235) *versus* 30PR (1017, SD = 276) (2 sample t-test: t_8_ = 3.325, *p* = 0.010, Figure 13c) and 5 times higher in 40CR (4095, SD = 929) *versus* 40PR (878, SD = 136) (2 sample t-test: t_9_ = 5.783, *p* < 0.0005, Figure 13d).

As with the two 12AL groups, the level of non-FAA were significantly different in 20CR compared to 20PR and 30CR to 30PR groups. Non-FAA in 20CR mice was 1.5 times lower than the 20PR (2 sample t-test: t_11_ = −4.702, *p* < 0.0005) and 1.6 times in the 30CR compared to 30PR (2 sample t-test: t_8_ = −4.976, *p* = 0.001). However at the end of the study the levels of non-FAA in the 40CR and 40PR were similar (7455, SD = 974 and 9532, SD = 1382 respectively, 2 sample t-test: t_9_ = −1.267, *p* = 0.237).

**Figure 14:**
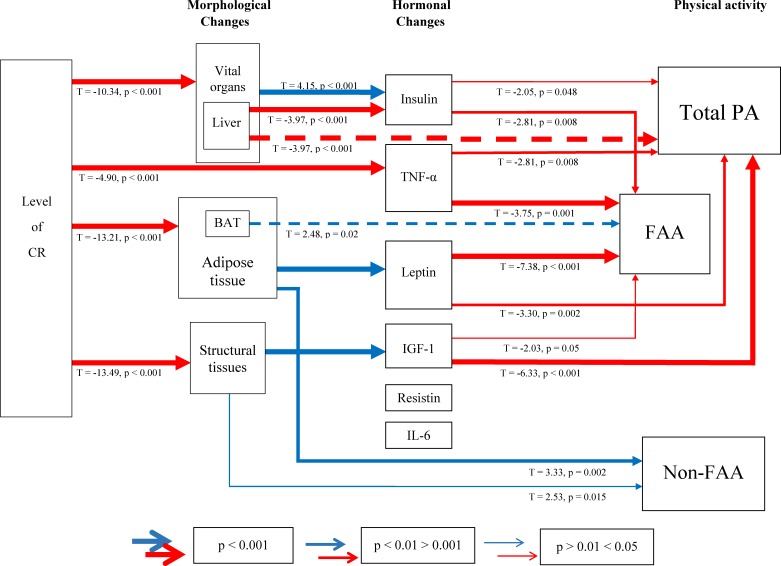
A schematic diagram depicting the interplay between morphological, hormonal changes and physical activity (PA) under calorie restriction (CR) PA was split into total daily PA, food anticipatory activity (FAA) and non-FAA. Relationships were generated using stepwise least squared multiple regression models. Positive relationships are shown in red and negative in blue. Increasing thickness in lines indicate increased significance. BAT = brown adipose tissue, TNF-α = tumor necrosis factor-α, IGF-1 = insulin-like growth factor-1, IL-6 = interleukin-6.

Amassing these results, the associations between the levels of CR, morphological and hormonal changes and their impact on PA are illustrated in Figure 14. Morphological changes in adipose tissues, structural tissues and vital organs impart changes in hormone levels which in turn, relay an effect on PA. In summary, leptin, insulin, TNF-α and IGF-1 levels are significantly negatively related to total PA and FAA. The levels of resistin and IL-6 were not related to PA and none of the hormones measured influenced non-FAA. We emphasize that the associations in this figure reflect correlations and cannot be assumed to be causal.

## DISCUSSION

### Calorie restriction

Mice are normally nocturnal, resting in the light phase and more active over the dark phase. This circadian behavior is primarily determined by the daily light-dark cycle, and is controlled by the master clock in the suprachiasmatic nucleus (SCN) [37, 63]. Other cues, such as food availability, are also capable of controlling sleep/wake behaviors in mammals. As with many CR studies (reviewed in [3]), our mice were fed once a day. To minimize disturbance weighing and feeding was carried out immediately before lights out, to coincide with the natural circadian arousal pattern. Early speculations on the mechanisms behind the impact of CR on lifespan hypothesized the pro-longevity effect was mediated by concomitant increases in spontaneous activity. Several studies in rats have shown that the age-related decline in activity is retarded by CR [48, 67].

While we found a decrease in total daily PA over the 3 months of study, this was apparent in all groups, and no overall difference in the level of daily PA was detected between the AL and CR mice at the end of study. However contradictory reports exist in the literature and some found an increase [34, 68] while others a decrease [35, 36, 69] in PA. Supporting our findings Cameron *et al* (2011) also found no difference in total levels of PA between the CR and AL mice, when CR was initiated at 14 months of age for 70 days [65]. Studies in non-human primates also found PA was not altered by CR [70], however studies on humans have reported decreased PA under CR [71, 72].

The change in the temporal patterning of PA throughout the day in response to CR, as found here, has been reported previously by several groups [32, 65, 73]. By fitting a Bayesian model Cameron *et al* (2011) found that, under CR, mice compensated for bouts of high activity (FAA) by prolonged phases of inactivity [65]. However, results here revealed that while many of the mice responded to high FAA with a lower non-FAA, this was not the case for all mice (3 in particular having high FAA and non-FAA). What was also immediately apparent was the high variation in levels of PA in response to the higher levels of CR. While it would be intuitive to suppose increased PA would result a higher loss of weight, no relationship was apparent between total daily PA and body composition.

We previously reported that these mice compensated for the reduced energy supply under CR with a preferential utilization of body fat [11]. While no relationship between total daily PA and fat mass or structural tissues was apparent here, FAA was strongly correlated with both these morphological changes. The level of body fat was also related to non-FAA. The level of body fat is proportionally related to the secretion of leptin, a key regulator of energy balance, signaling a reduction in energy intake and an increase in energy expenditure [74]. While reduced levels of leptin, as a consequence of CR, might be predicted to be associated with a decrease in total PA, as a means of saving energy, hypoleptinemia, as found in the most restricted mice, was associated with increased total daily activity, particularly FAA. The role of leptin in PA control can be clearly defined in the leptin deficient *ob/ob* mouse which is phenotypically obese and hypoactive [75]; characteristics which can be reversed by administration of leptin [76]. However, leptin control over PA may be more complicated with contrasting effects of leptin reported on locomotor activity and FAA [58]. Physiological levels of leptin resulted in an increase in locomotor activity which contrasted with a deficiency of leptin, which appeared to suppress FAA in *ob/ob* mice [58]. In this study we found that, in addition to leptin, insulin, TNF-α and IGF-1 were strongly related to total PA and FAA and like leptin none of these other hormones influenced non-FAA.

While the roles of leptin and insulin as regulators of energy balance are well documented, less has been reported regarding the relationship between TNF-α and IGF-1 and energy balance. TNF-α, a key inflammatory cytokine, also classed as an adipokine, is becoming known for its role in energy homeostasis [77, 78]. Elevated TNF-α was previously reported to have an important role in the depressed appetite symptomatic of infections and in conditions associated with disease such as cancer cachexia [79]. Recently a role of TNF-α in the lipopolysaccharide induced circadian effect on locomotor activity rhythm was suggested [80]. TNF-α levels in the mice studied here were reduced with CR [15]. While we show here that leptin, insulin, TNF-α and IGF-1 were associated with FAA a number of other hormones we did not measure have also been implicated in FAA control, eg. norepinephrine, dopamine and ghrelin. Regulation of FAA is clearly complex and not fully understood, and the complete ablation of FAA has not been observed in any gene knockout model studied to date.

FAA was first reported by Curt Paul Richter in rats in 1922 [81], since then it has been well documented in many mammals, fish and birds [39] with mice shown to anticipate as many as 4 of 6 daily meals [66]. Foraging for food in the wild is necessary for survival and FAA may reflect increased activity [41]. We found that after 3 months FAA comprised almost 40% of daily activity in mice under 40CR. A 4 fold increase in light phase activity in mice under a similar level of CR mice was previously reported [33]. The control of FAA appears to be distinct from the SCN; mice with lesions of the SCN and those lacking BMAL1, a critical gene for circadian rhythm, display a robust FAA response under restricted feeding [82, 83]. Several food-entrainable oscillators have been discovered elsewhere in the brain and throughout the body, ie clock gene rhythms in stomach, intestine, pancreas, liver, adrenal, heart, lung, muscle and others all realign to the daily rhythm of food intake [38, 84]. Here we found that mice subjected to higher levels of CR responded more acutely and exhibited greater intensity of FAA.

When faced with limited energy availability it would seem logical that energy saving mechanisms such as reduced basal metabolism and increases in the use of torpor would be activated. We found both total daily PA and FAA were correlated to T_b_, in the mice that exhibited higher FAA had lower mean and minimum T_b_ ie torpor (T_b_ <31^°^C). Note that over much shorter timescales (minutes rather than days), however, these associations were reversed and mice which had lower body temperature were less active [20]. At the scale of days rather than minutes, our data agreed with previous work showing that mice faced with high foraging costs (running) were more likely to employ torpor than mice exposed to low foraging costs [85]. Our results imply that energy saved by use of torpor could be cancelled out by the increased energy expended on FAA. Interestingly a positive association was found between BAT and FAA. BAT plays important role in the regulation of energy balance and CR has been shown to inhibit BAT thermogenesis and retard the age-related decline in mitochondrial function of BAT [86-88].

### Protein restriction

Several studies have reported increases in longevity following PR (or specific amino acids such as tryptophan [89] or methionine [90]) and have implicated the specific reduction in protein to underlie the beneficial effect of CR. Our results found no indication of an effect of PR on total daily PA, FAA or non-FAA. While restricted feeding is a powerful zeitgeber and one of a limited number of external cues that can entrain the circadian clock and override the nocturnal activity rhythm [91], we found no evidence of changes in circadian rhythms in mice fed a restricted protein diet. Over a complex series of experiments, Mistlberger and colleagues sought to define macronutrient cues for FAA [92]. Their earlier work had shown mice developed nutrient-dependent FAA to sweetened meal [93], however, chronically protein deprived mice did not show anticipation for a single protein meal (this was also true for carbohydrate and fat). More recent research, however, has concluded glucose availability can produce FAA in rats [94]. Results here show FAA was due to restriction of calories alone with no evidence that equivalent levels of PR increased FAA.

Although in general the response to PR differed from the response to CR, one area of similarity was in the relationship between fat mass, leptin and total PA levels. Under both CR and PR the relationships were both negative (Figures 3 and 12). This may reflect different processes in the different treatments. Under CR there was a major effect of the level of CR on body fatness [11] which led to very low leptin levels [17], and this was strongly related to the level of FAA (Figure 7c) thus driving the overall relationship between leptin and total PA (Figure 3a). In contrast the mice under PR did not show any FAA (Figure 13) hence the relationship between overall activity and leptin levels (Figure 12) could not be underpinned by an association between FAA and body fat/leptin levels. We have previously shown that individual variation in PA levels in C57BL/6 mice can be a factor driving individual variability in weight and fat gain, with low activity hence predisposing to weight and fat gain and hence increased leptin levels [95]. Hence the relationship within the mice under PR may have come about because of this association.

In conclusion, no differences in average daily PA were found between CR treatment groups. Beneath this lack of difference however, there were tremendous differences in the temporal patterning of PA, in particular the balance between the levels of FAA and non-FAA. While most mice studied here appeared to show compensatory reduction in non-FAA when FAA was high, this was not always the case. Although there was a large variation in activity responses to CR, the strong linear trend observed in FAA in relation to the level of restriction was consistent with the linear increase in lifespan [96]. This change in activity is unlikely to be mimicked in humans under CR treatment. Hence, if it plays a role in the life extending impact of CR such benefits will be unlikely to translate to humans. Separately, CR and exercise have been shown to increase mean lifespan in rodents, but CR and not exercise can impact on maximum longevity [46, 97]. While both strategies induce a leaner body mass (less fat mass) with similar health benefits observed, there is no additive effect and the combination of CR and exercise did not extend lifespan further than CR alone [48, 97, 98]. While many similarities exist, there are clearly fundamental differences between these two interventions. Voluntary exercise improved heart health *via* reductions in production of hydrogen peroxide [99]. Yet, despite decreased levels of IGF-1 and reduced DNA damage in exercised *ad libitum* fed rats compared to sedentary controls and exercising animals which had increased insulin sensitivity relative to their CR counterparts, exercise is unable to mimic the life extending effect of CR [100-101].

## EXPERIMENTAL PROCEDURES

### Animals

The rationale and design of the study has been detailed previously [11]. All procedures were reviewed and approved by University of Aberdeen ethical approval committee and carried out under a Home Office issued license compliant with the Animals (Scientific Procedures) Act 1986. In brief male C57/BL6 mice (Charles River, Ormiston, UK) were acclimated for 6 weeks prior to implantation of transmitters at 12 weeks of age, allowing adequate recovery time prior to experimentation. A number of baseline measurements, including dual X-ray absorptiometry (DXA) for body composition, glucose tolerance tests (GTT) and resting metabolic rate, were carried out at 17-18 weeks old. Over the baseline period all animals** were provided with *ad libitum* access to water. Open source diet (D12450B, Research Diets, NJ, USA) containing 20% protein, 70% carbohydrate and 10% fat (by energy) was provided in the 12 hours of darkness only.

CR or PR was begun at 20 weeks of age, approximately equivalent to early adulthood of humans. In the CR study all mice continued to be fed D12450B with restriction levels set at 10%, 20%, 30% and 40% (referred to as 10CR, 20CR, 30CR and 40CR) of individual baseline food intake For the PR study diets were designed to match the reduced protein level of the 20CR, 30CR and 40CR, ie protein content was equivalent to 16, 14 and 12% protein (made up by increased carbohydrate) (D13020201, D13020202 and D13020203 respectively, Research Diets, NJ, USA). These are referred to as 20PR, 30PR and 40PR. Mice may compensate for the reduced protein intake by overeating; this was prevented by feeding a fixed weight of food equivalent to their own individual baseline intake on D12450B (20% protein). All four diets were isocaloric (3.8kcal/g) and the duration of restriction phase was 3 months.

With mice naturally nocturnal, 24hr access to food may also invoke unnatural overeating leading to obesity. This can be problematic when used as controls against restricted feeding [3, 102]. Therefore an additional group where access to food was limited to the 12 hours of darkness was used as a control and referred to as 12AL. Food intake of the 12AL and 24AL groups did not differ significantly [11]. To minimize light phase disturbances mice were fed once per day, immediately prior to lights out and food was removed at the onset of light phase. Both CR and PR studies utilized a control 12AL group with animals treated identically. Mice were killed on day 90 of restriction between 1400 and 1700h to minimize any circadian effects on hormone levels and body composition [11].

### VitalView^™^

Physical activity (PA) (and core body temperature) were measured using the VitalView^™^ telemetry and data acquisition system (MiniMitter, OR, USA). The transmitters, implanted intraperitoneally, are unrestrictive allowing free movement of the animals. Minute by minute recordings are transmitted *via* an ER-4000 receiving platform and VitalView^™^ software was used to acquire data (MiniMitter, OR, USA). For a full description of the system refer to [103]. Due to a malfunction of 2 transmitters post implantation *n* = 8 for all groups bar 30CR where *n* = 6. Animals were undisturbed except for feeding, weighing and routine checks.

### Statistical analysis

Statistical analyses were performed using the IBM SPSS Statistics package 23 and Minitab version 17. Data was checked for normal distribution using the Kolmogorov-Smirnov test and log transformed where applicable. The current analysis concerns data summarized over time periods of days, and major subcomponents of whole days. For an analysis of activity of the same mice at the time scale of minutes refer to [19]. The minute by minute data summed over 24h was called total daily activity. The sum over the 2 hr prior to feeding was called food anticipatory activity (FAA) and this was subtracted from the total activity to give non-FAA. These three types of activity were analyzed separately. The effect of the level of CR over the course of the study was explored using general linear model (GLM) with repeated measurements (RM). Where significance was achieved between treatment groups post hoc Tukey tests were used. One way ANOVA was used for comparison between groups at the end of the study. Paired or 2 sample t-tests were used where appropriate. Quadratic equations were fitted to the data and the time to reach the nadir in activity and the initial rate of change from the onset of restriction were derived from these fitted equations. The relationships between total daily PA, FAA and non-FAA over the last 20 days of the experiment with body composition, circulating hormone levels and corresponding body temperatures measured at the end of the experiment we determined using least squares single and multiple regression analysis (LSR), eliminating non-significant terms using a backward elimination stepwise procedure. Linear regression analyses were verified by exploration of the diagnostic plots of residuals against fitted values. For detailed protocols of body composition, hormone and body temperature measurements please refer to [11, 15, 17] respectively. All data are shown as mean ± standard deviation (SD) with results considered statistically significant at p ≤ 0.05.

## SUPPLEMENTARY MATERIAL FIGURES


