# Supplementary material for: The effects of graded levels of calorie restriction: V. Impact of short term calorie and protein restriction on physical activity in the C57BL/6 mouse
Source: Oncotarget. 2016 Mar 17;7(15):19147–70. doi: 10.18632/oncotarget.8158 (PMC4991372; doi:10.18632/oncotarget.8158)
Supplement: Supplementary file 1 [file oncotarget-07-19147-s001.pdf]

The effects of graded levels of calorie restriction: V. Impact of short term calorie and protein restriction on physical activity in the C57BL/6 mouse

Supplementary Material

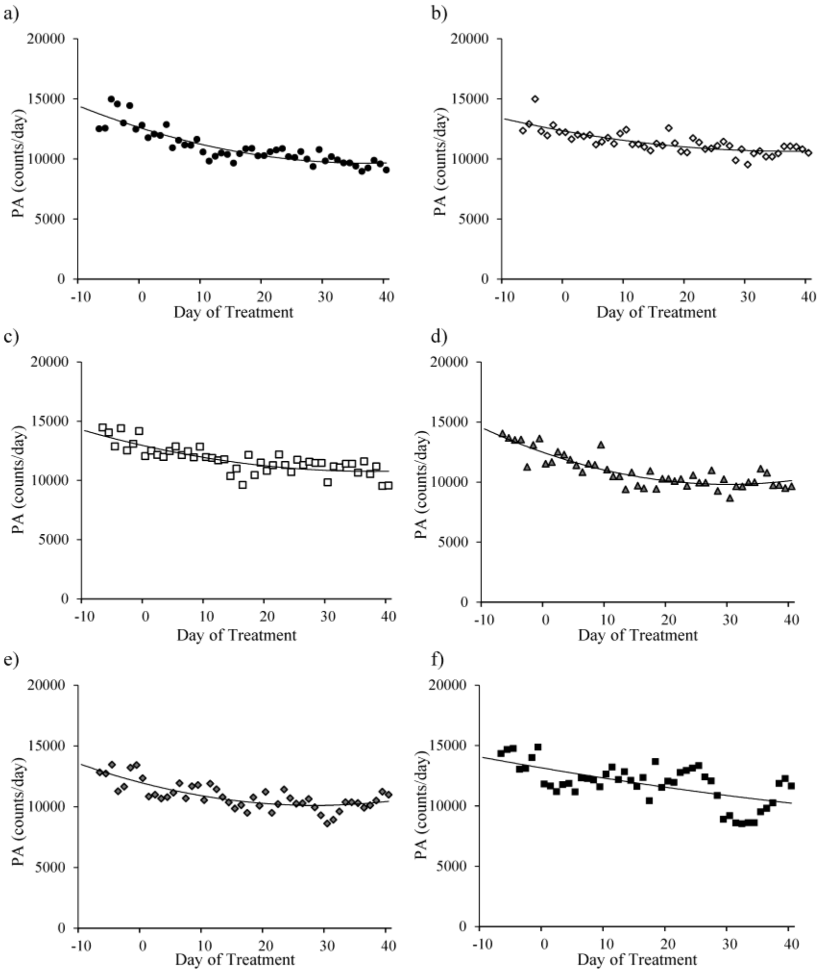

Supplementary Figure 1. Mean daily physical activity counts (PA) over the first 40 days of calorie restriction (CR). The baseline period is represented by negative days and CR began at day 0. a) & b) Control animals were fed *ad libitum* for 24 and 12 hrs (24AL and 12AL respectively). c) – f) 10CR, 20CR, 30CR and 40CR which were restricted by 10, 20, 30, and 40% respectively. Polynomial curves were fitted and detailed statistic shown in Table 1.

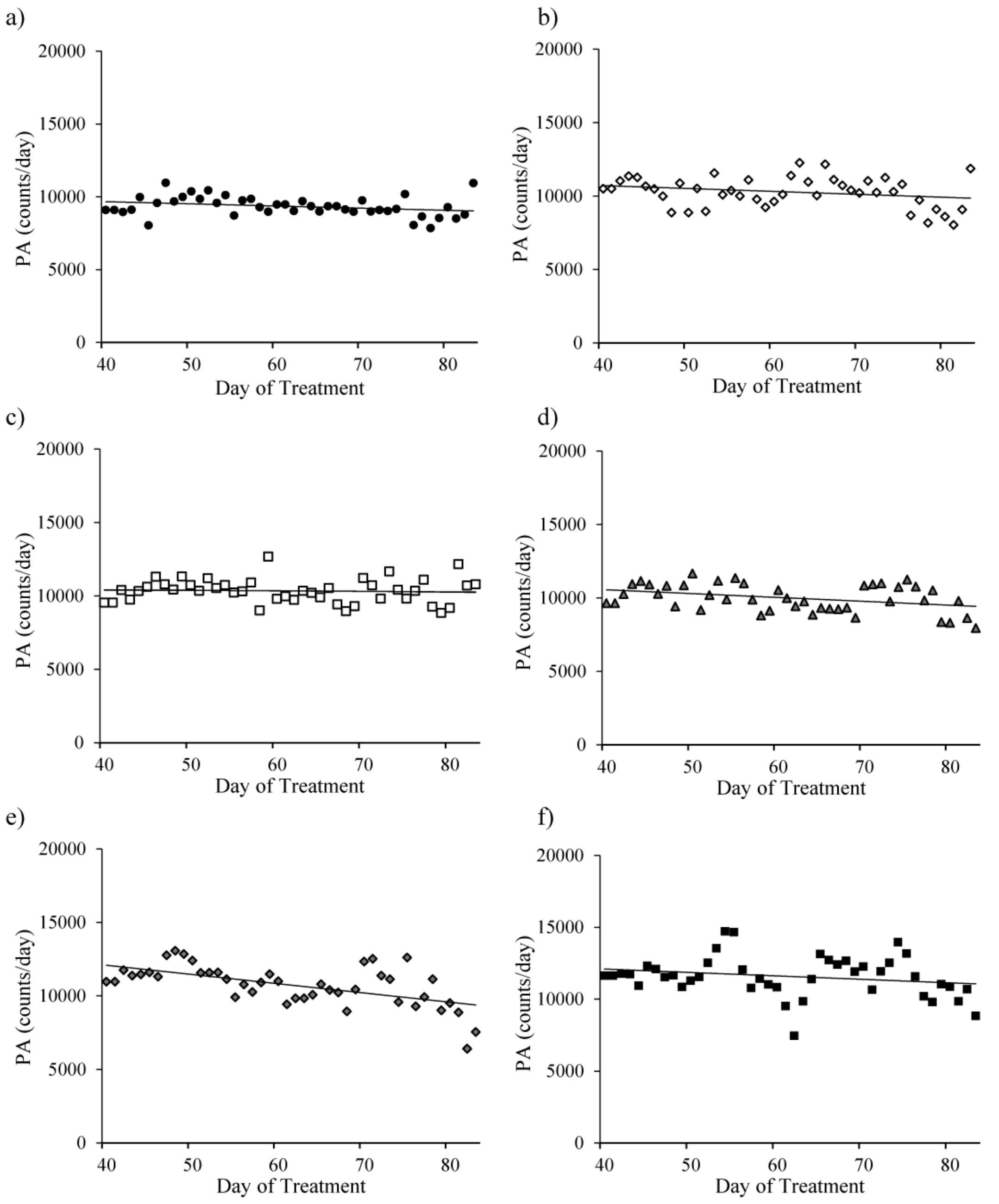

Supplementary Figure 2. Mean daily physical activity counts (PA) over the final 40 days of calorie restriction (CR). a) & b) Control animals were fed *ad libitum* for 24 and 12 hrs (24AL and 12AL respectively). c) – f) 10CR, 20CR, 30CR and 40CR which were restricted by 10, 20, 30, and 40% respectively.
